# Supplementary material for: DNA Methylation Signatures Predict Cytogenetic Subtype and Outcome in Pediatric Acute Myeloid Leukemia (AML)
Source: Genes (Basel). 2021 Jun 10;12(6):895. doi: 10.3390/genes12060895 (PMC8229099; doi:10.3390/genes12060895)
Supplement: Supplementary file 1 [file genes-12-00895-s001.zip › Krali_Palle_SupplementaryFigures_R1.pdf]

Supplementary Figures

DNA methylation signatures predict cytogenetic subtype and outcome in pediatric acute myeloid leukemia (AML)

Olga Krali [1+], Josefine Palle [1,2+*], Christofer L. Bäcklin [3], Jonas Abrahamsson [4], Ulrika Norén-Nyström [5], Henrik Hasle [6], Kirsi Jahnukainen [7], Ólafur Gísli Jónsson [8], Randi Hovland [9], Birgitte Lausen [10], Rolf Larsson [3], Lars Palmqvist [11], Anna Staffas [11], Bernward Zeller [12] & Jessica Nordlund[1*]

[1] Department of Medical Sciences, Molecular Precision Medicine and Science for Life Laboratory, Uppsala University, Sweden
[2] Department of Women's and Children's Health, Uppsala University, Sweden
[3] Department of Medical Sciences, Cancer Pharmacology and Computational Medicine, Uppsala University, Sweden
[4] Department of Pediatrics, Queen Silvia Children's Hospital, Gothenburg, Sweden
[5] Department of Clinical Sciences, Pediatrics, Umeå University Hospital, Umeå, Sweden
[6] Department of Pediatrics, Aarhus University Hospital, Denmark
[7] Children's Hospital, Helsinki University Central Hospital, Helsinki, and University of Helsinki, Finland
[8] Department of Pediatrics, Landspitali University Hospital, Reykjavík, Iceland
[9] Center of Medical Genetics and Molecular Medicine, Haukeland University Hospital, Norway
[10] Department of Pediatrics and Adolescent Medicine, Rigshospitalet, University of Copenhagen, Denmark
[11] Department of Clinical Chemistry and Transfusion Medicine, University of Gothenburg, Sweden
[12] Division of Paediatric and Adolescent Medicine, Oslo University Hospital, Oslo, Norway
* Authors to whom correspondence should be addressed
[+] Equal contribution

```
hyperparameters = {'NeuralNetwork': {'NeuralNetwork__epochs':[100,200],
                                     'NeuralNetwork__batch_size':[8, 16],
                                     'NeuralNetwork__activation' : ['relu', 'sigmoid'],
                                     'NeuralNetwork__dim1' : [100, 200]},

                   'NearestCentroid':{'parameters': None},
                   'KNeighborsClassifier':{'KNeighborsClassifier__estimator__n_neighbors': [5,10,15,20],
                                           'KNeighborsClassifier__estimator__weights':['uniform', 'distance'],
                                           'KNeighborsClassifier__estimator__algorithm': ['auto', 'ball_tree', 'kd_tree', 'brute'
                   'SVC':{'SVC__estimator__kernel':['linear', 'poly', 'rbf', 'sigmoid'],
                          'SVC__estimator__degree':[3,4],
                          'SVC__estimator__C':[1, 0.1, 0.01],
                          'SVC__estimator__gamma':['scale','auto']},
                   'GaussianProcessClassifier':{'parameters': None},

                   'DecisionTreeClassifier':{'DecisionTreeClassifier__estimator__criterion':['gini', 'entropy'],
                                             'DecisionTreeClassifier__estimator__max_features':['auto', 'sqrt', 'log2'],
                                             'DecisionTreeClassifier__estimator__splitter':['best','random']},

                   'RandomForestClassifier':{'RandomForestClassifier__estimator__n_estimators':[10, 50, 100, 200],
                                             'RandomForestClassifier__estimator__criterion':['gini', 'entropy'],
                                             'RandomForestClassifier__estimator__max_features':['auto', 'sqrt', 'log2']},


                   'AdaBoostClassifier':{'AdaBoostClassifier__estimator__n_estimators':[10, 50, 100],
                                         'AdaBoostClassifier__estimator__learning_rate':[0.01, 0.1, 1]},


                   'GaussianNB':{'parameters': None}
                   }
```

Figure S1. Hyperparameters used to optimize for each of the nine classification models.

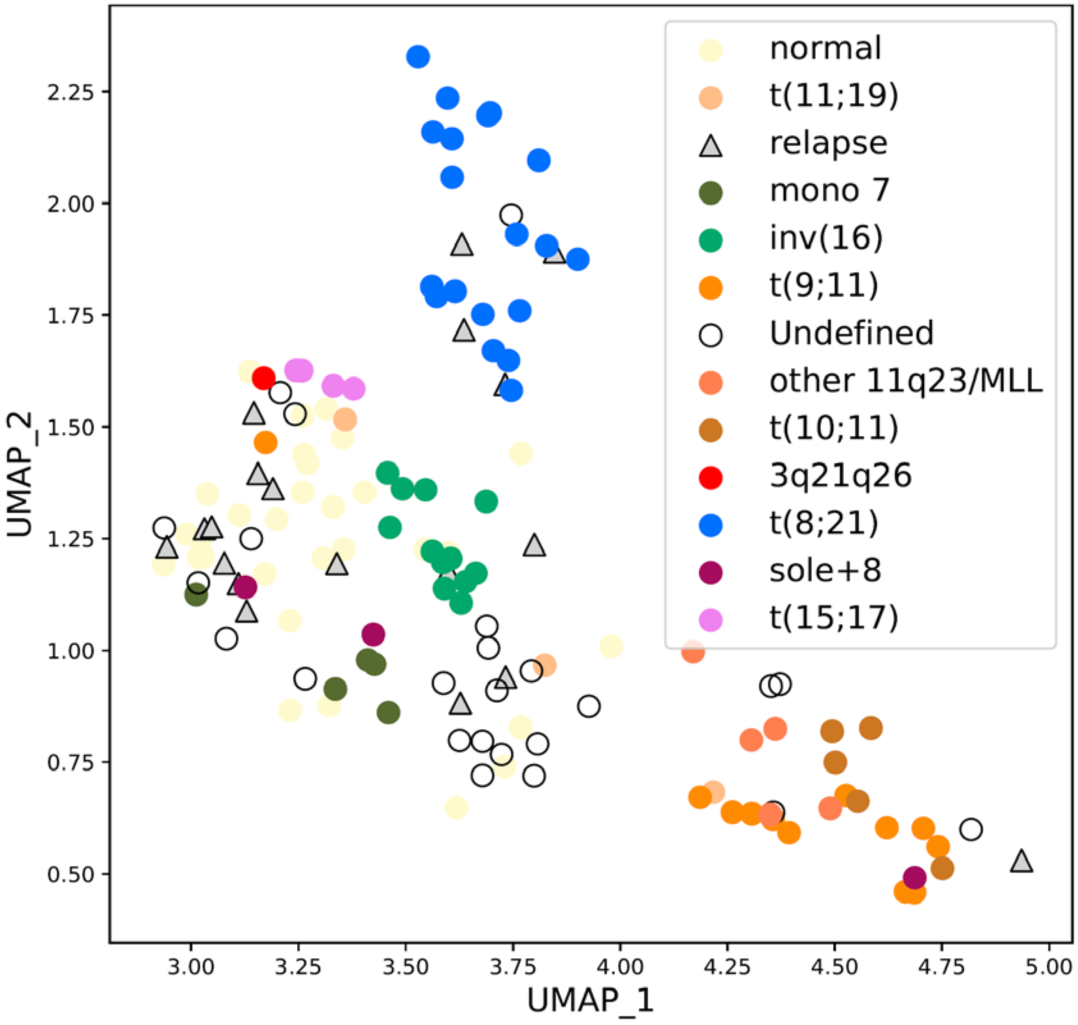

Figure S2. UMAP representation of the complete 450k dataset and patients labelled by cytogenetic subtype.

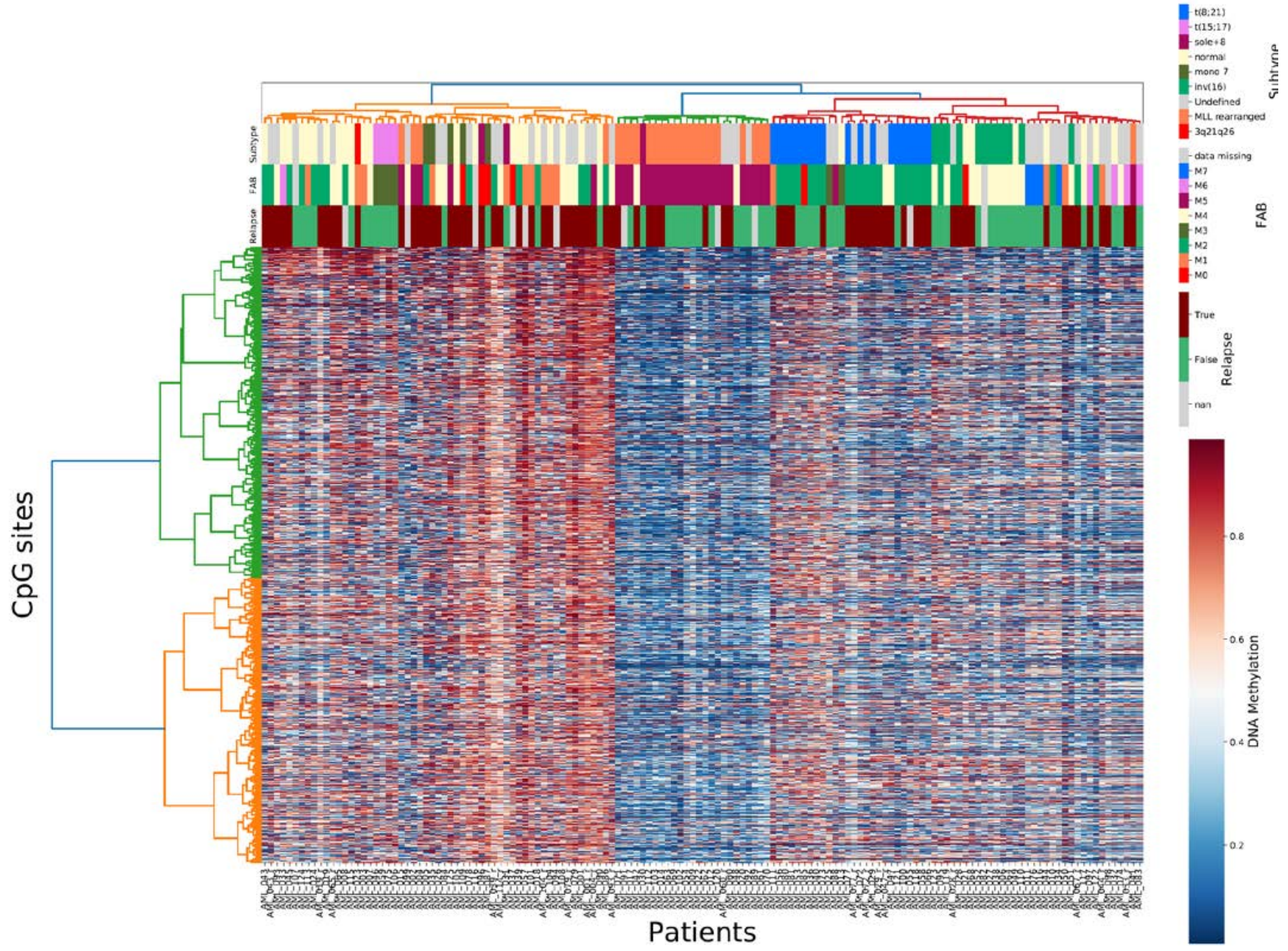

Figure S3. Heatmap of the 10,000 most variable CpG sites ordered by hierarchical clustering.

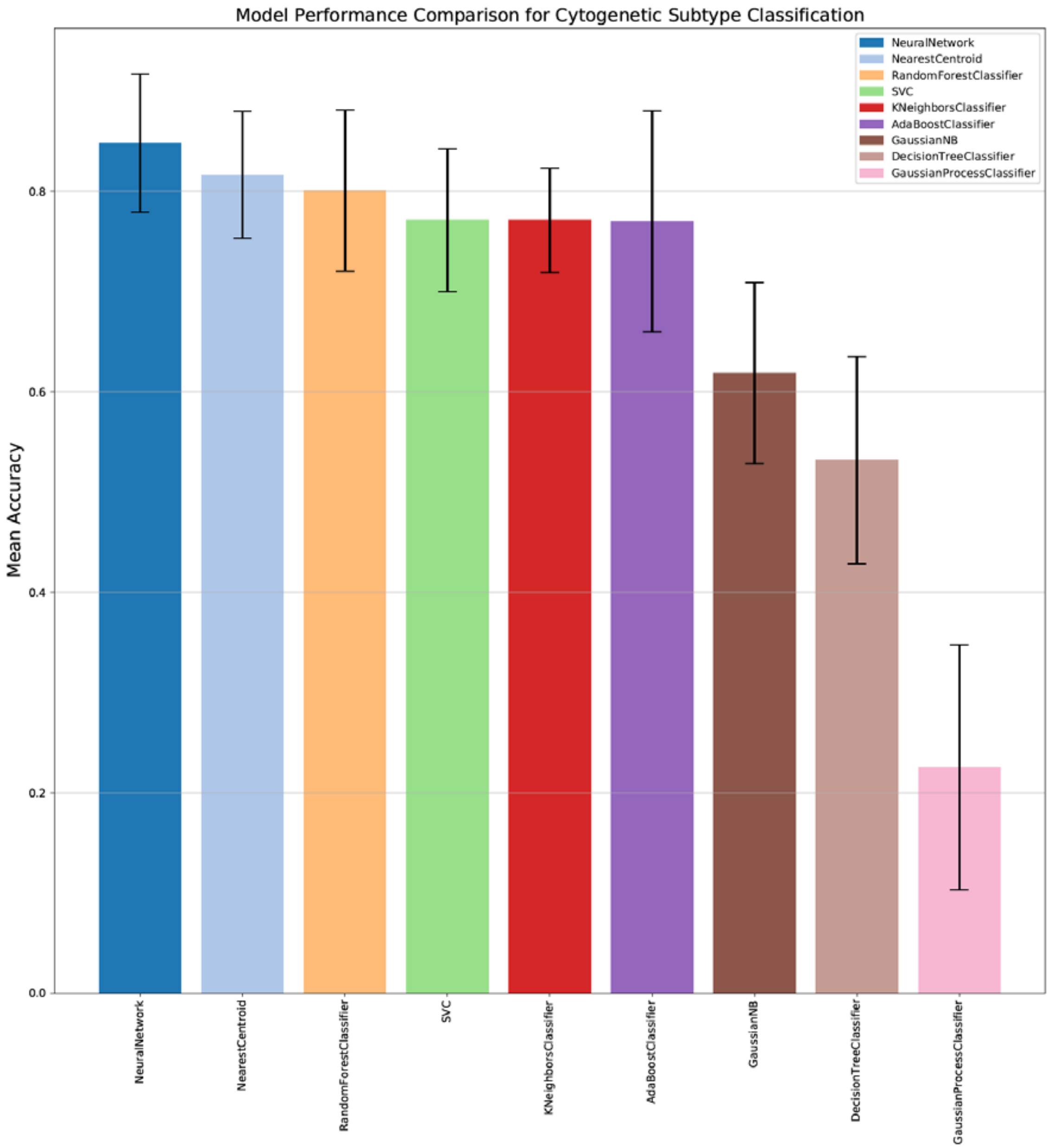

Figure S4: Model performance accuracy scores for the nine optimized classification models. Mean accuracy of the classifier and standard deviation (error bars) is shown on the y-axis. The nine classification models are plotted along the x-axis.

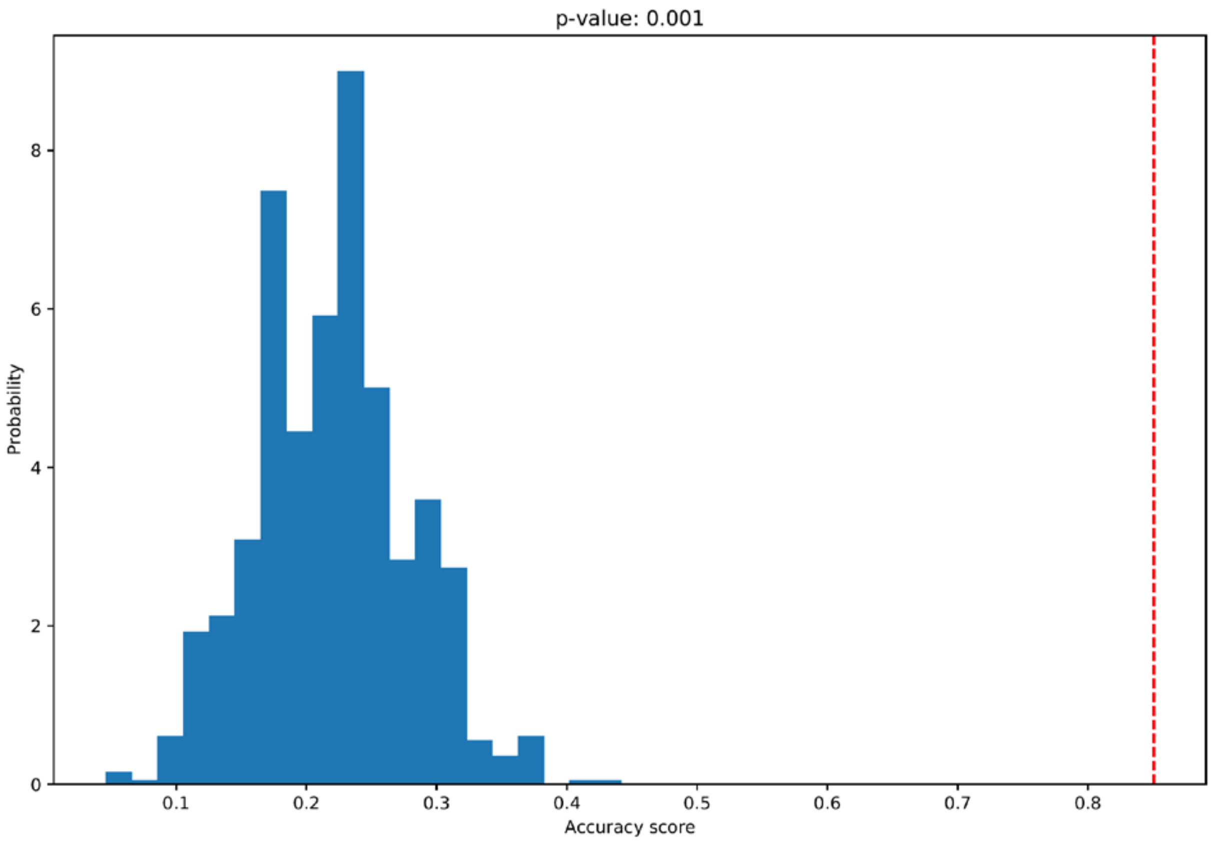

Figure S5: Permutation analysis scores for Neural Network Classification model for 1000 random permutations. The red dashed line demonstrates the model score on the test data. The p-value from the Wilcoxon signed-rank test is shown above the plot.

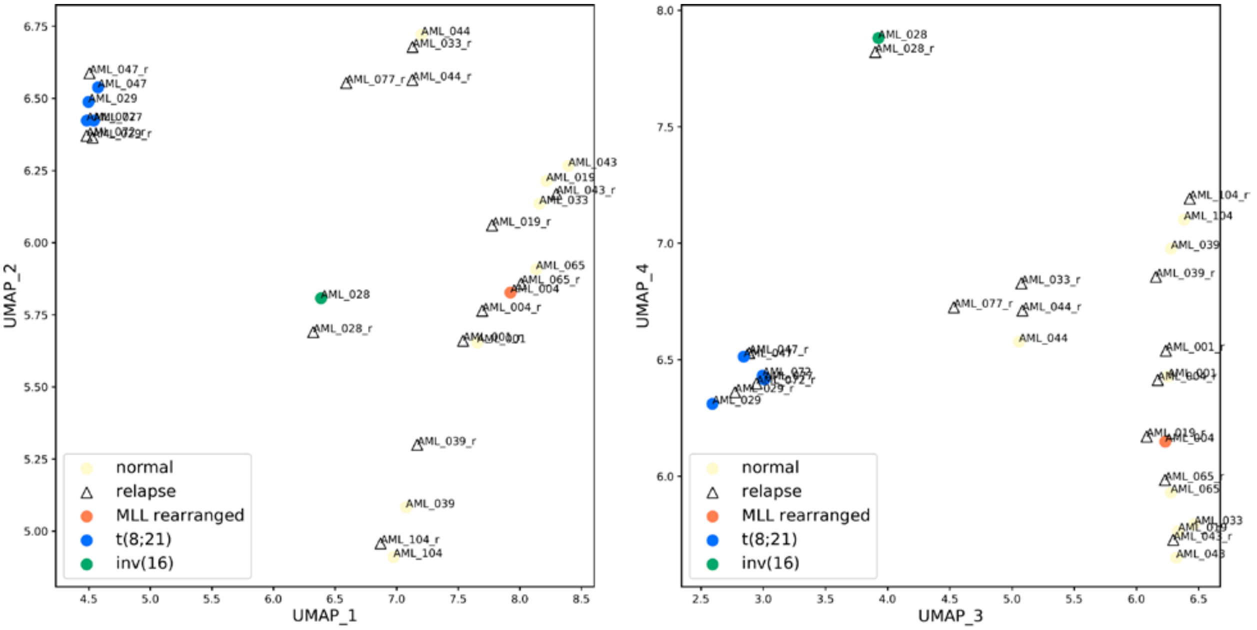

Figure S6: UMAP representation of the 14 pairs of diagnostic and relapsed samples. The clustering is based on the 1300 selected CpGs and samples are labeled by the sample ID (AML ID) and color coded by diagnostic cytogenetic subtype.

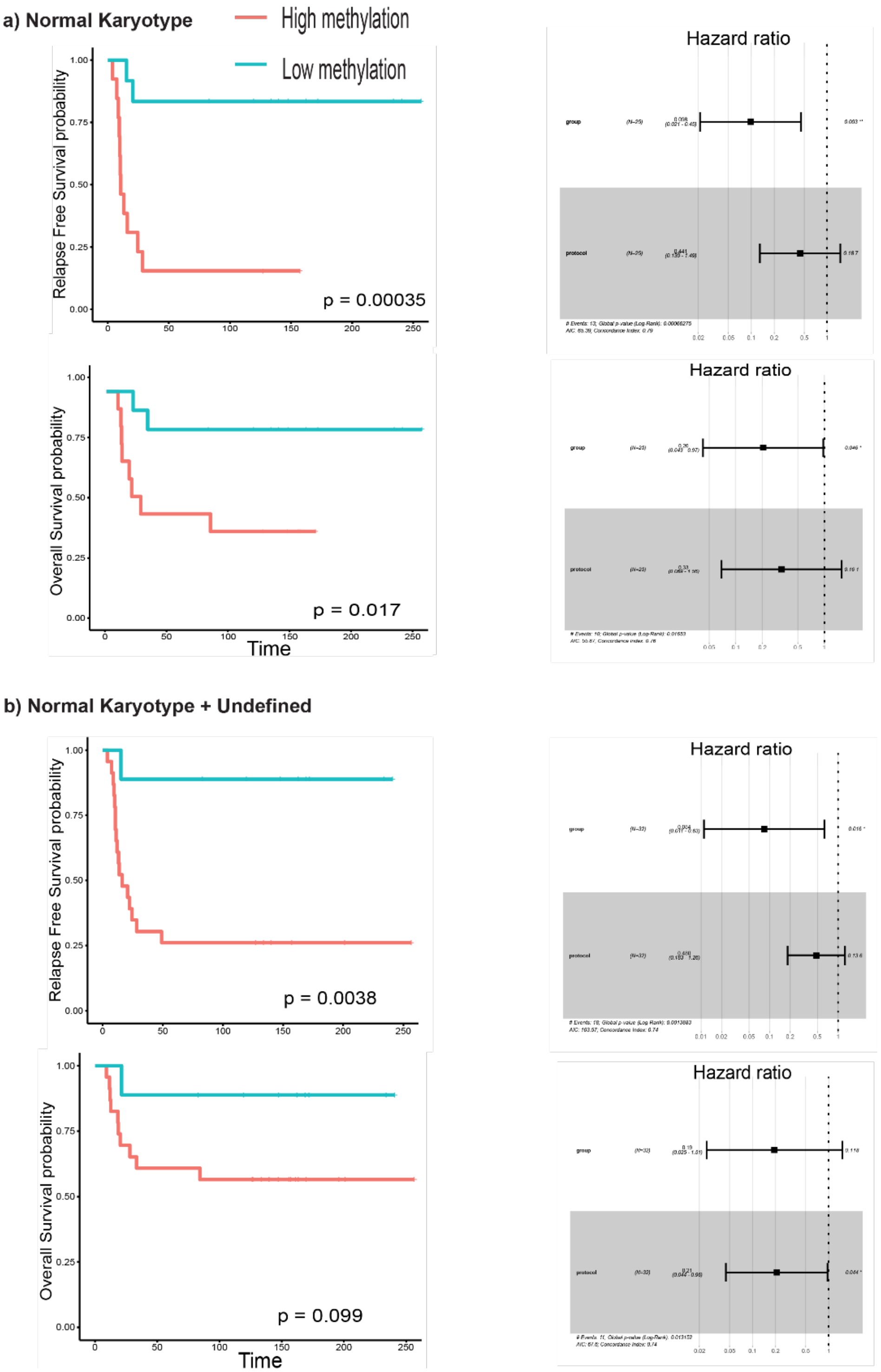

Figure S7. Survival analysis of the NK subtype patients treated on the NOPHO-93 or NOPHO-2004 protocols. A) Kaplan-Meier curves and cox regression analysis for relapse-free and overall survival based on 25 NK of confirmed diagnostic subtype (diagnostic-known) and B) in the diagnostic-known NK samples in addition to the seven DNA-methylation predicted samples of undefined cytogenetics. Results from the Cox Regression analysis is plotted to the right. Methylation group (group) and treatment protocol (NOPHO-93 vs NOPHO-2004, i.e. protocol) are included as covariates.

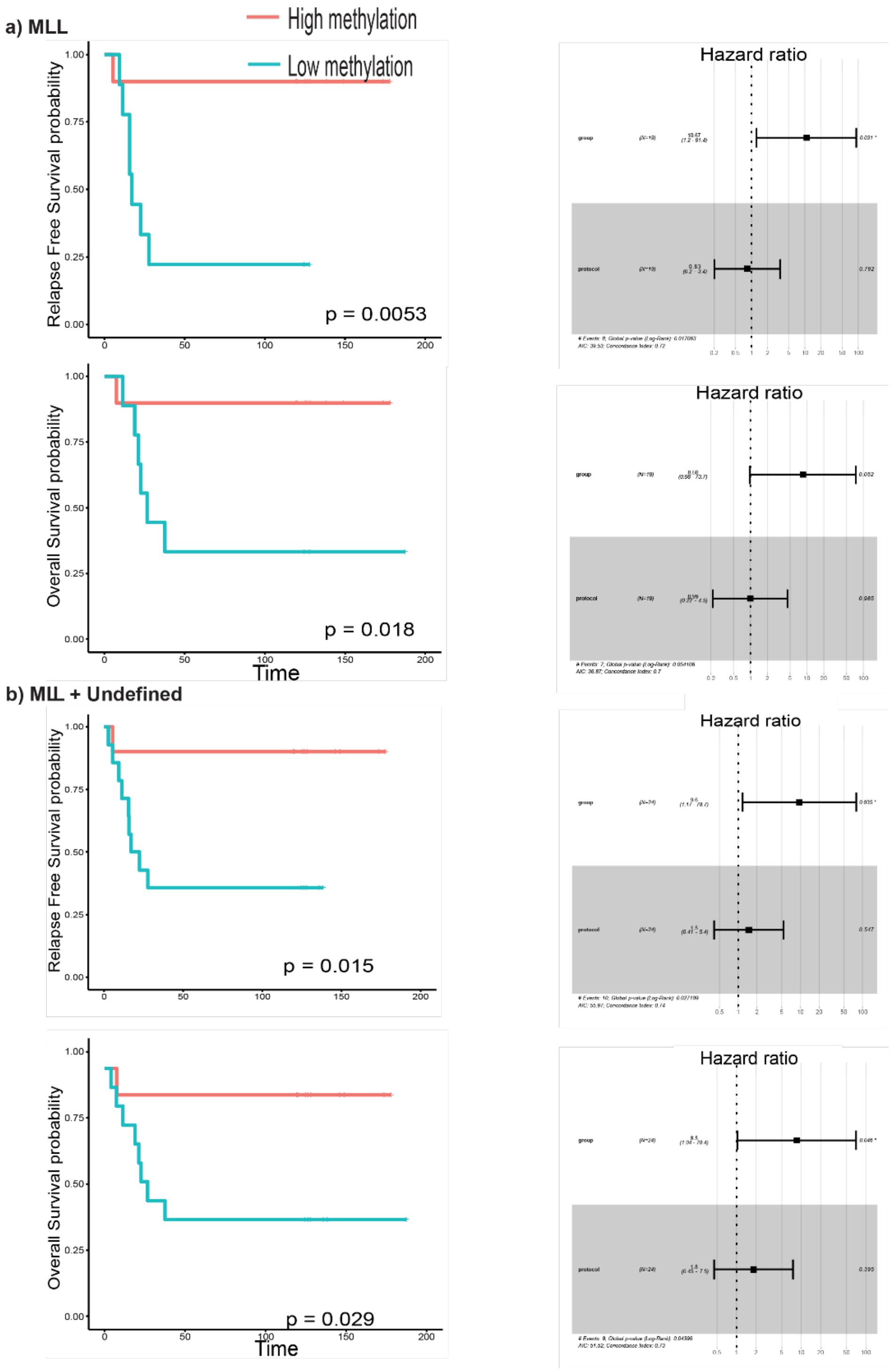

Figure S8. Survival analysis of the *MLL*/*KMT2A*-rearranged patients treated on the NOPHO-93 or NOPHO-2004 protocols. A) Kaplan-Meier curves and cox regression analysis for relapse-free and overall survival based on 19 *MLL*/*KMT2A*-rearranged cases with confirmed diagnostic subtype (diagnostic-known) and B) in the diagnostic-known *MLL*/*KMT2A*-rearranged cases in addition to the five DNA-methylation predicted samples of undefined cytogenetics. Results from the Cox Regression analysis is plotted to the right. Methylation group (group) and treatment protocol (NOPHO-93 vs NOPHO-2004, i.e. protocol) are included as covariates.

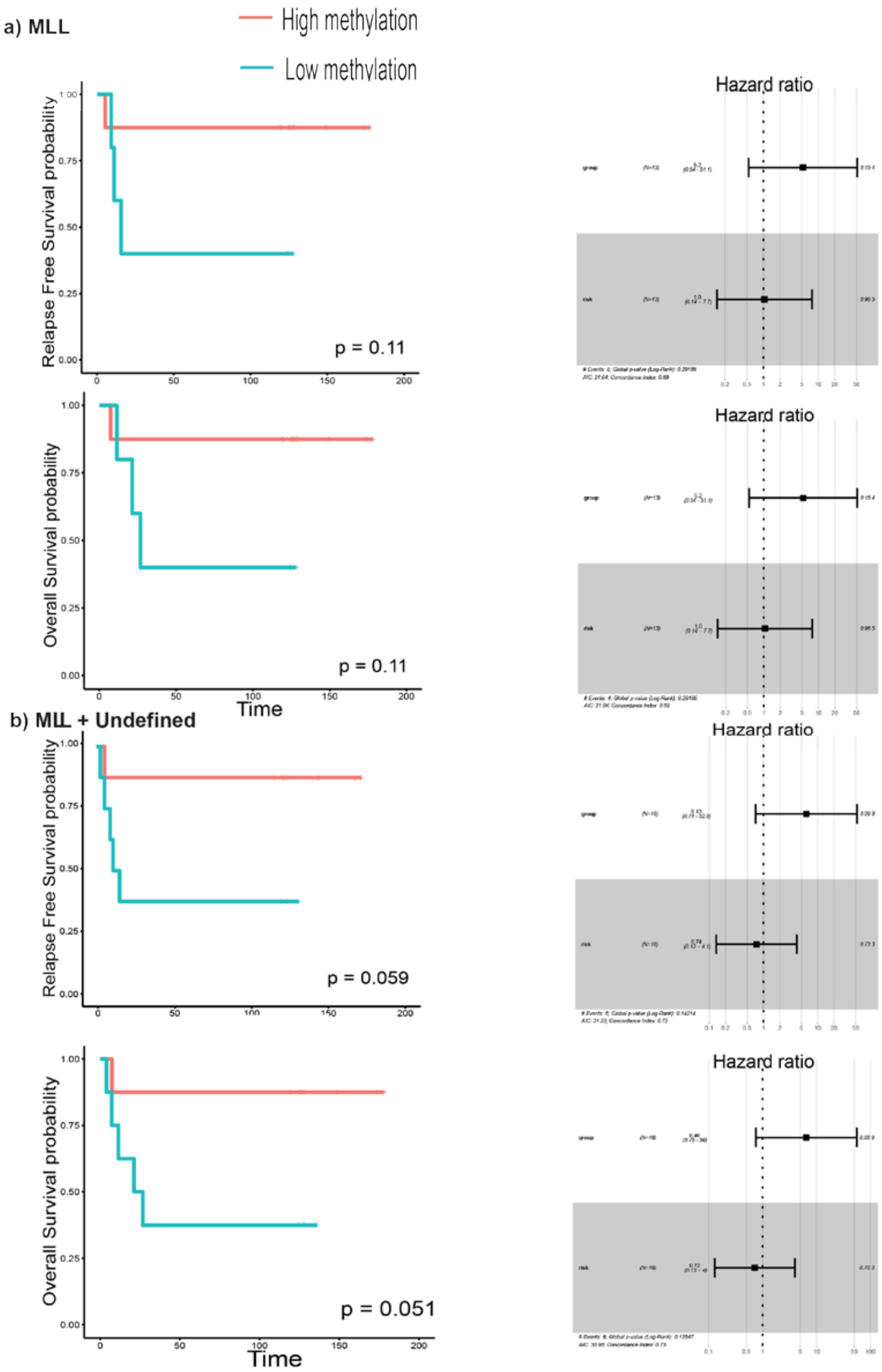

Figure S9. Survival analysis of the *MLL*/*KMT2A*-rearranged patients treated on the NOPHO-2004 protocol. A) Kaplan-Meier curves and cox regression analysis for relapse-free and overall survival based on 13 MLL-rearranged cases with confirmed diagnostic subtype (diagnostic-known) and B) in the diagnostic-known *MLL*/*KMT2A*-rearranged cases in addition to the three DNA-methylation predicted samples of undefined cytogenetics. Results from the Cox Regression analysis is plotted to the right. Methylation group (group) and risk group (standard vs high risk, i.e. risk) are included as covariates.
